# Prevalence of Menstrual Dysfunction and Hormonal Contraceptive Use Among Elite Female Athletes from Different Sports in Germany

**DOI:** 10.1186/s40798-025-00845-6

**Published:** 2025-05-07

**Authors:** Elisabeth M. Kirschbaum, Katharina Fischer, Dorothee Speiser, Franziska Lautenbach, Friedemann Schwenkreis, Anne Dathan-Stumpf, Kirsten Legerlotz

**Affiliations:** 1 Movement Biomechanics, Institute of Sport Science, Humboldt-Universität zu Berlin, Berlin, Germany; 2 Research Group Elite Female Athletes, Institute for Applied Training Science, Leipzig, Germany; 3 Sports Psychology, Faculty of Sports Science, University of Leipzig, Leipzig, Germany; 4 Department of Gynecology with Breast Center, Charité - Universitätsmedizin Berlin, Berlin, Germany; 5 Sports Psychology, Institute of Sport Science, Humboldt-Universität zu Berlin, Berlin, Germany; 6 Business Information Systems, Baden-Wuerttemberg Cooperative State University Stuttgart, Stuttgart, Germany; 7 Department of Obstetrics, University Hospital of Leipzig, Leipzig, Germany; 8 Department of Movement and Training Science, Institute of Sport Science, University of Wuppertal, Wuppertal, Germany

**Keywords:** Menstrual cycle, Women, Sports disciplines, Oligomenorrhea, Amenorrhea, Prevalence, Gynecological health

## Abstract

**Background:**

While a regular menstrual cycle is an indicator of good health, menstrual dysfunction (MD) and wrong beliefs regarding hormonal contraceptives (HC) are quite common among elite female athletes and threaten their health. This study aimed to (1) identify the prevalence of current and lifetime MD in various sports disciplines, (2) investigate variables that are associated with the prevalence of MD, (3) study the current practice of HC use among elite German female athletes.

**Methods:**

584 German elite female athletes (mean 20.7 ± 4.9 years) from 64 different sports completed an online questionnaire to assess gynecological health characteristics, history of MD, and the use of HC.

**Results:**

Sixty-nine percent of athletes not using HC reported a regular menstrual cycle, while oligomenorrhea was currently reported in 13%, secondary amenorrhea in 8%, primary amenorrhea in 2% and polymenorrhea in 8%. The current prevalence of primary amenorrhea differed between sports disciplines (*p* = 0.002), while oligomenorrhea (*p* = 0.828) and secondary amenorrhea (*p* = 0.848) did not. The lifetime prevalence of primary amenorrhea (10%) and oligomenorrhea (74%) differed significantly between sports disciplines (*p* < 0.001 and *p* = 0.001), while secondary amenorrhea (40%) did not (*p* = 0.298). The current and lifetime prevalence of primary amenorrhea was higher in aesthetic sports disciplines, while the lifetime prevalence of oligomenorrhea was higher in endurance disciplines. Factors associated with lower prevalences of MD were menstrual cycle tracking (*p* < 0.001), higher gynecological age (*p* < 0.001), regular gynecological health screenings (*p* = 0.008), and a diagnosed eating disorder (*p* = 0.037). Twenty-nine percent of these elite athletes used HC, of whom 15% claimed to use HC as a treatment for MD.

**Conclusion:**

Elite female athletes from a variety of sports disciplines, not just from endurance and aesthetic sports, are at high risk of developing MD. Given the high percentage of athletes using HC to treat MD, educating athletes, coaches, others from the support team and parents about the risk and prevention of MD and the effects of HC in the context of elite sports may improve gynecological health among elite athletes.

**Supplementary Information:**

The online version contains supplementary material available at 10.1186/s40798-025-00845-6.

## Key Points

- Elite female athletes from all sports disciplines, not just from endurance and aesthetic sports, are at risk of developing menstrual dysfunction (MD). The prevalence of current oligomenorrhea and secondary amenorrhea did not differ between sports disciplines.
- Regular gynecological health screenings and menstrual cycle tracking were associated with a lower prevalence of MD among elite athletes.
- Our results indicate that education programs for athletes, coaches, others from the support team and parents and the implementation of structures, such as annual MD risk factor screenings or access to sports-affiliated gynecologists, are needed to improve gynecological health among elite female athletes.

**Supplementary Information:**

The online version contains supplementary material available at 10.1186/s40798-025-00845-6.

## Background

Menstrual dysfunctions (MD), such as oligomenorrhea (duration between menstrual bleedings > 35 days) or secondary amenorrhea (absence of menstrual bleedings for > 3 months after menarche) are frequently observed in female athletes, particularly in leanness sports in which endurance or aesthetic movements determine success [1]. While many female athletes and their coaches are still unaware of this, the relationship between MD and intense physical activity has already been investigated for more than 50 years [2–4]. MD can result from low energy availability (LEA) [5], which can be caused intentionally by insufficient energy intake, resulting for example from disordered eating, or unintentionally by failure to increase the energy intake to match the training load [6]. While there can be many reasons for LEA and the associated weight changes, predisposing factors are the athletes’ body image and the social environment created by coaches or teammates [7]. LEA can result in significant negative health and performance consequences, also known as Relative Energy Deficiency in Sport (REDs) [8]. REDs due to problematic LEA may include e.g. decreased bone mineral density, depression, impaired energy metabolism, impaired growth, and negative effects on performance, such as impaired recovery, reduced training adaptation, and decreased athlete availability [8]. Even 10 days of severe LEA can reduce muscle protein synthesis [9], while only four days of severe LEA can negatively impact the hormonal regulation of the menstrual cycle (MC) [10].

Although it has been known for more than five decades that MD in female athletes is a health issue that must be taken seriously [4], to date it is still insufficiently addressed in female elite sports. Despite a regular MC being a vital sign of health in female athletes not using hormonal contraceptives (HC) [11], it is rarely systematically documented in athlete monitoring systems or discussed between athletes and coaches. Sports associations rarely provide programs and guidelines or initiate research activities on female health concerns [12], and coaches and female athletes prioritize other competing interests, e.g., training or sleep, over education about female health concerns [13]. This is partly because the MC is still perceived as a taboo topic among athletes and coaches [13–15] but also because of a lack of knowledge about the MC [13, 14]. As a result, MD is often considered to be a normal response to training among elite athletes, coaches, and medical support teams in leanness sports, and female athletes feel faced with the dilemma of choosing between a healthy MC and performance [13, 15]. Indeed, recent studies show a prevalence of MD among elite female athletes between 24% in Switzerland [16], 33% in Finland [17], and up to 51% in Denmark [18].

The actual prevalence of MD might be even higher than the reported numbers, as relying solely on MC length to identify MD leaves several subtle disorders, such as anovulation or luteal phase deficiency, undetected [19, 20]. Another factor is the use of HC, as especially the use of oral contraceptive pills could mask obvious and easily detectable MD. HC use is common among elite female athletes, as numbers from Australia (33%) [21], Switzerland (45%) [16], the UK (50%) [22], and Denmark (57%) [18] show. Moreover, some female athletes see HCs as an opportunity to regain a regular MC [16, 21, 22] and state “to initiate a menstrual bleeding” as a reason for HC use. Pharmacological treatment, such as HC, for MD could be considered if non-pharmacological treatment options have proved ineffective [11, 23]. However, when using HC as the only treatment for MD, REDs can further progress as no modifications are made to the underlying LEA, e.g., by adjusting diet or training volumes [11, 23]. Also, the use of HC limits the ability to use the MC as a health indicator [11, 21]. A widely mentioned solution for the treatment of MD and REDs is multidisciplinary teams, including sport psychologists, nutritionists and gynecologists [8, 11, 13, 15]. Yet, it has been shown that only 4% of elite female athletes in Sweden and Norway have access to gynecologists within their sports medical staff [14].

To date, there are no data on the prevalence of MD and the frequency of HC use among elite female athletes in Germany. Since the general framework for elite sport is unique in each nation, it seems to be important to assess national data. Moreover, previous studies have mainly focused on MD in endurance and aesthetic sports. Due to the increasing professionalization in many types of female sports, such as e.g. team sports, and the associated increasing physiological and psychological demands on female athletes, which may have an impact on the prevalence of MD, it seems important to assess MD in a range of different sports, to identify those most at risk. Furthermore, little is known about factors that can possibly contribute to improving gynecological health among female athletes, e.g., gynecological health screenings and MC tracking. It is still unclear whether there are differences in the frequency of HC use in terms of sports disciplines, but also from a sociocultural perspective. Moreover, it seems to be important to understand athletes’ reasons and expectations for using HCs. An improved understanding can provide a solid framework for MC and HC education and enables more effective advice for HC use.

Therefore, the first aim of this study was to identify the prevalence of current and lifetime MD in elite female athletes of various sports disciplines hypothesizing that the prevalences are higher in endurance and aesthetic sport disciplines. Secondly, we aimed to investigate variables that are associated with the prevalence of MD such as the frequency of gynecological health screenings, MC tracking, or intentional weight changes, hypothesizing that gynecological health screenings and MC tracking will decrease MD. Thirdly, we wanted to describe the frequency of HC use, reasons for use as well as perceived positive and negative side effects.

## Methods

### Participants

**Fig. 1:**
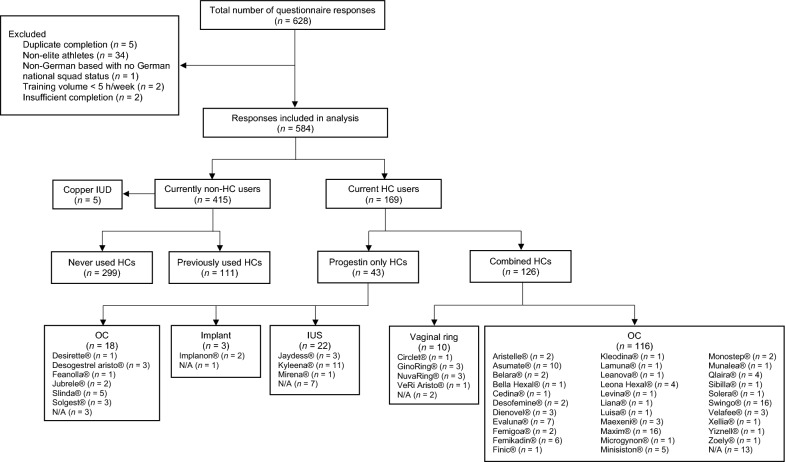
Overview and HC characteristics of the female athletes included in the study (*n* = 584). Frequency of non-HC users and type, delivery method, and frequency of HC use. HC, hormonal contraceptives; IUD, intrauterine device; OC, oral contraceptives; IUS, intrauterine system; N/A, not applicable

Between August 2022 and February 2023, German elite female athletes were recruited by national sports federations, Athleten Deutschland e.V. (Germany’s independent Athletes’ Association), and social media. In this study, an elite athlete was defined as one who (1) has a national squad status in Germany or (2) competes internationally or at the highest national competition level. For inclusion in the study, participants were required to complete the full questionnaire and to train currently at least 5 h per week. All participants provided written informed consent, and written parental consent was obtained for those under the age of 16, in accordance with the Regulation 2016/679 (GDPR). The study was approved by the ethics review board of the Institute for Applied Training Science (ER_2022.01.17_12). Data from 628 German female elite athletes were collected. After exclusion of datasets due to e.g., duplicates or non-elite status (Fig. 1), a total of 584 were included in the final analysis.

Athletes from 64 different sports disciplines participated and were divided into seven groups for further analysis, following previously published approaches [24, 25]: (1) endurance (*n* = 154), (2) power (*n* = 87), (3) aesthetic (*n* = 43), (4) weight class (*n* = 48), (5) ball games (*n* = 135), (6) technical (*n* = 111), and (7) anti-gravity (*n* = 6) (Table 1). The main characteristics for categorization into the groups were (1) high volume aerobic training and high metabolic requirements, (2) maximization of strength and power leads to performance improvement, (3) judges assess athletes’ performance, (4) categories based on body mass, (5) a ball is involved and the aim is to score more goals or points than the opponent, (6) special technical skills combined with a piece of specialized equipment, and (7) gravity has to be overcome during performance.

**Table 1:** Classification of sports into different sports discipline groups (*n* = 584)

Endurance (*n* = 154)	Power (*n* = 87)	Aesthetic (*n* = 43)	Weight class (*n* = 48)	Ball games (*n* = 135)	Technical (*n* = 111)	Anti-gravity (*n* = 6)
Biathlon (*n* = 16)	Alpine skiing (*n* = 37)	Figure skating (*n* = 6)	Boxing < 92 kg (*n* = 1)	Badminton (*n* = 7)	Archery (*n* = 7)	Climbing (*n* = 1)
Mountainbike endurance (*n* = 3)	Mountainbike trial (*n* = 1)	Artistic cycling (*n* = 3)	Judo < 78 kg (*n* = 23)	Basketball (*n* = 2)	Curling (*n* = 1)	Ski jumping (*n* = 5)
Canoeing (*n* = 11)	Bobsleigh (*n* = 1)	Artistic gymnastics (*n* = 3)	Ju-Jutsu < 70 kg (*n* = 4)	Field hockey (*n* = 44)	Fencing (*n* = 42)	
Cross-country skiing (*n* = 25)	Breaking (*n* = 1)	Artistic swimming (*n* = 1)	Karate < 68 kg (*n* = 1)	Handball (*n* = 12)	Pentathlon (*n* = 11)	
Race walking (*n* = 1)	Hammer throw (*n* = 1)	Dancing (*n* = 1)	Lightweight rowing (*n* = 7)	Lacrosse (*n* = 2)	Sailing (*n* = 11)	
Running ≥ 800 m (*n* = 5)	Inline speed skating (*n* = 1)	Diving (*n* = 1)	Taekwondo < 72 kg (*n* = 7)	Para ball games (*n* = 5)	Shooting (*n* = 39)	
Nordic combined (*n* = 3)	Javelin (*n* = 1)	Rhythmic gymnastics (*n* = 22)	Weight lifting < 87 kg (*n* = 2)	Rugby (*n* = 9)		
Orienteering (*n* = 5)	Judo > 78 kg (*n* = 3)	Trampoline (*n* = 5)	Wrestling (*n* = 3)	Soccer (*n* = 11)		
Para canoeing (*n* = 1)	Ju-Jutsu > 70 kg (*n* = 1)			Softball (*n* = 2)		
Para swimming (*n* = 2)	Shot put (*n* = 3)			Tennis (*n* = 3)		
Road cycling (*n* = 16)	Ski cross (*n* = 10)			Ultimate frisbee (*n* = 4)		
Rowing (*n* = 55)	Ski freestyle (*n* = 3)			Volleyball (*n* = 34)		
Speed skating ≥ 1500 m (*n* = 1)	Snowboard (*n* = 2)					
Swimming ≥ 400 m (*n* = 7)	Speed skating < 1500 m (*n* = 1)					
Triathlon (*n* = 3)	Sprint (*n* = 2)					
	Surfing (*n* = 1)					
	Swimming < 400 m (*n* = 10)					
	Track cycling (*n* = 8)					

### Questionnaire

For data collection, we used an anonymous online questionnaire (SoSci Survey GmbH, Munich, Germany) in German and English. Prior to the release, a group of athletes (*n* = 9) and gynecologists (*n* = 4) provided feedback on the questionnaire. The questionnaire was part of a larger project and contained six parts: demographics, gynecological health, knowledge about the MC/HC/REDs, performance during the MC, communication about the MC, and preferred ways of receiving information on the MC's effects. In order to focus on prevalence of MD and HC use, in this paper we present the demographic and gynecological health data, while the results of the other parts are described elsewhere.^1^ The questions on demographics included general personal data, such as age, body mass, height, and sports-related information about sports discipline, competition level, professional status, weekly training volume, gender of the main coach, gender distribution of the support team and experience in the main sport. The gynecological health data part contained questions about pre-existing gynecological conditions, the frequency of gynecological check-ups, urinary stress incontinence, stress fractures, diagnosed eating disorders, and intentional weight changes with more than 2 kg weight gain or loss in the last two years and reasons for intentional weight changes. In addition, all participants were questioned on menstrual cycle characteristics such as age at menarche, perceived MC-related symptoms and time of the symptoms, MC length, bleeding duration, frequency of period pain, intake of painkillers during menstruation and impairment of everyday life due to periods, oligomenorrhea and secondary amenorrhea including also the time of occurrence, as well as pregnancies, and births. HC users were asked about the contraception method and the brand, side effects, perceived disadvantages and advantages, as well as reasons for use. All participants were asked about the use of HCs in the past. The questionnaire used can be found in the electronic supplementary material.

### Data Analysis

Duplicates were removed by checking the respondent-generated personal code and demographic information. To analyze open-ended questions, a quantitative content analysis was conducted. First, categories were pre-defined based on the answers given, and second, EMK and KF independently assigned the answers to the various categories. Disagreements between the researchers were resolved through discussion until a consensus was reached.

Gynecological age was calculated from the current age minus the age at menarche [26]. The prevalence of primary amenorrhea was given if the athlete did not report the onset of menarche while being ≥ 16 years when completing the questionnaire [27]. The lifetime prevalence for primary amenorrhea was assessed by analyzing the age at menarche of all athletes and including also all athletes with a current primary amenorrhea.

According to Janni et al. [27] polymenorrhea was defined as MC length ≤ 24 days, oligomenorrhea as MC length between 36 and 90 days, and secondary amenorrhea as MC length > 90 days. The prevalences were assessed based on self-reported information on the average intervals in days between menstrual bleedings of current non-HC users. The participants were classified into the MD group if they currently had primary amenorrhea, oligomenorrhea, or secondary amenorrhea as defined by the REDs indicators [8]. The lifetime prevalence of oligomenorrhea was calculated from the athletes who currently had oligomenorrhea and the athletes who had never used HC previously and reported an interval of more than five weeks (> 35 days) between their menstrual bleedings in the past. The lifetime prevalence of secondary amenorrhea was calculated from the athletes who currently reported secondary amenorrhea and the athletes who have not used HC previously and reported an interval of more than three months (> 90 days) between their menstrual bleedings in the past.

### Statistical Analysis

All analyses were carried out using MS Excel (Microsoft Corp, Redmond, WA, USA), IBM SPSS Statistics for Windows, version 29 (IBM Corp., Armonk, NY, USA), and RapidMiner Studio version 10.3.001 (Altair Engineering Inc., Troi, MI, USA). Descriptive data were generated for all variables. The Shapiro–Wilk test was used to test if the data was normally distributed. Data of none of the variables were normally distributed and therefore, the Mann–Whitney test and Kruskal Wallis test were performed to assess group differences. To compare proportions between groups, we used the Chi-Square test with Fisher’s exact test, and Bonferroni-adjusted post-tests were used to examine pair-wise group differences. Binary logistic regression analysis was used to identify variables that were independently associated with MD. Variables with *p* < 0.10 in the bivariate analysis of unsquared eta coefficient or Cramer’s, were included in the multivariate analysis. For this reason, the variables competition level (*p* = 0.984), weight changes (*p* = 0.221) and stress fractures (*p* = 0.226) were excluded. Age was excluded from the logistic regression due to its high collinearity to gynecological age (*r* = − 0.876). Odds ratios and 95% confidence intervals were estimated. The importance of attributes was derived from the logistic regression model based on the odds ratios which resulted in the following importance ranking: diagnosed eating disorder, gender of the main coach, gender distribution of the support team, leanness sports, frequency of gynecological health screenings, professional status, weekly training volume, BMI and gynecological age. Since the logistic regression showed a relatively low sensitivity, the relevance of the attributes with respect to MD was verified using a calculation of the corresponding information gain [28]. The extracted ranking of the attributes based on the information gain was: gynecological age, BMI, gender distribution of the support team, gender of the main coach, weekly training volume, frequency of gynecological health screening, professional status, sports discipline, and diagnosed eating disorder. The low sensitivity of the logistic regression and the significant difference in the sequence of relevance of the information gain ranking compared to the logistic regression ranking of attributes, leads to the conclusion that logistic regression is a suboptimal approach to determine the statistical relationship between MD and the measured variables. A simple linear function is not suitable to separate the cases from a mathematical point of view. Alternatively, a Classification and Regression Tree (CART) model based on the information gain as the split criterion was used to estimate MD based on the measured variables. Rather than using the tree model to predict MD based on data of the future, we extracted rules from the model to gain insights into the multivariate interdependencies associated with MD in the currently collected data. Hence, the same data were used for training as well for testing to determine the quality of the model. All data are presented as mean ± SD, frequencies, or prevalence. A *p*-value ≤ 0.05 was considered significant.

## Results

### Participants Characteristics

The athletes were on average 20.7 ± 4.9 years old, 170.0 ± 7.9 cm tall, had a mean BMI of 22.0 ± 2.4 kg/m^2^, and trained on average 17.9 ± 7.1 h per week. The average training experience in the sport was 11.1 ± 5.1 years. Of all athletes, 75% (*n* = 435) were competing internationally, and 31% (*n* = 182) were professional athletes. Fifteen percent (*n* = 88) were coached by females, and in 26% (*n* = 149) the gender distribution in the athletes’ support team was balanced or consisted of more females. The athletes grouped into seven sports disciplines differed in age, height, body mass, BMI, self-reported weekly training volume, experience in the main sport, competition level, proportion of professional status, the gender of the main coach, and the gender distribution in the support team (Table 2).

**Table 2:** Demographic data from participants classified into sports disciplines (*n* = 584)

	Endurance	Power	Aesthetic	Weight class	Ball games	Technical	Anti-gravity	*p*-value
Age, y	20.4 ± 2.4 ^a,b*^(*p* = 0.027)	20.3 ± 4.4 ^b^(*p* = 0.001)	18.5 ± 4.8 ^c^(*p* = 0.027), ^d^(*p* = 0.010), ^e^(*p* = 0.023)	23.0 ± 4.8 ^c*, d^(*p* = 0.002), ^e^(*p* = 0.002), ^f^(*p* = 0.001)	21.1 ± 5.3 ^a^(*p* = 0.010), ^b^(*p* = 0.002)	21.1 ± 5.7 ^a^(*p* = 0.023), ^b^*(p* = 0.002)	21.0 ± 3.2	< 0.001
Height, cm	171.6 ± 8.3 ^a*,b^(*p* = 0.009), ^e^ (*p* = 0.036), ^f*,g^(*p* = 0.045)	168.2 ± 6.6 ^c*,d*^	165.0 ± 6.7 ^c*,d*^	167.4 ± 7.1 ^c^(*p* = 0.009), ^d^(*p* = 0.002)	173.1 ± 8.6 ^a*,b^(*p* = 0.002), ^e^(*p* = 0.008), ^f*,g^(*p* = 0.027)	168.8 ± 6.1 ^c^(*p* = 0.036), ^d^(*p* = 0.008)	162.7 ± 5.2 ^c^(*p* = 0.045), ^d^(*p* = 0.027)	< 0.001
Body mass, kg	64.2 ± 8.8 ^a*,g^(*p* = 0.039)	64.4 ± 8.9 ^a*,g^(*p* = 0.049)	53.7 ± 8.3^b*,c*,d*,e*,f*^	62.7 ± 7.7^a*^	66.1 ± 7.8 ^a*,g^(*p* = 0.005)	63.9 ± 9.0^a*^	54.0 ± 3.5 ^c^(*p* = 0.039), ^d^(*p* = 0.005), ^f^(*p* = 0.049)	< 0.001
BMI, kg/m^2^	21.8 ± 2.4 ^a*^	22.7 ± 2.4 ^a*^	19.6 ± 2.0 ^b*,c*,d*,e*,f*^	22.3 ± 1.9 ^a*^	22.1 ± 2.0 ^a*^	22.4 ± 2.8 ^a*^	20.4 ± 1.1	< 0.001
Internationally competing, n (%)	119 (77%)	61 (70%)	34 (79%)	42 (88%)	87 (64%)	87 (78%)	5 (83%)	0.025
Professional athletes, n (%)	52 (34%)	42 (48%)^d*^	11 (26%)	16 (33%)	26 (20%) ^f*^	31 (28%)	4 (67%)	< 0.001
Experience in the main sport, y	9.6 ± 4.5 ^b*, d*^	10.3 ± 4.9 ^b*^	12.0 ± 4.5	14.8 ± 5.2 ^c*,e*,f*^	12.2 ± 4.8 ^c*^	10.9 ± 5.5 ^b*^	8.8 ± 4.0	< 0.001
Self-reported total weekly training volume, h	17.5 ± 5.1 ^a*,d*^	20.1 ± 5.9 ^a^(*p* = 0.010), ^d*,e*^	28.9 ± 9.8 ^c*,f^(*p* = 0.010), b*,d*,e*	17.4 ± 4.2 ^a*,d^(*p* = 0.017)	14.4 ± 5.2 ^a*,b^(*p* = 0.017), ^c*, f*^	16.9 ± 7.8 ^a*,f*^	20.1 ± 7.2	< 0.001
Male main coach, n (%)	141 (92%) ^a*^	78 (90%) ^a*^	14 (33%) ^b*,c*,d*,e*,f*,^ ^g^(*p* = 0.003)	43 (90%) a*	115 (85%) a*	99 (89%) ^a*^	6 (100%) ^a^(*p* = 0.003)	< 0.001
Dominance of males in support team, n (%)	119 (77%) ^a*,f^(*p* = 0.001)	81 (93%) ^a*,b^(*p* = 0.001), ^d*,e*^	6 (14%) ^b*,c*,d*, e*,f*,g*^	43 (90%) ^a*^	98 (73%) ^a*,f*^	82 (74%) ^a*,f*^	6 (100%) ^a*^	< 0.001

BMI, body mass index; HC, hormonal contraceptive; *n* (%), sample size (percentage of)

Data are presented in the form: mean ± SD or *n* (%). ^a^different from aesthetics, ^b^different from weight-class, ^c^different from endurance, ^d^different from ball games, ^e^different from technical, ^f^different from power, ^g^different from anti-gravity, *indicates a significant difference with *p* < 0.001

### Overall Gynecological Health Characteristics

The average age at menarche was 13.3 ± 1.5 y, and the average gynecological age was 7.6 ± 4.9 y. Almost every fifth athlete (19%) reported stress urinary incontinence, every third athlete (33%) reported intentional weight changes, 5% reported a diagnosed eating disorder, and 8% reported diagnosed stress fractures (Table 3). HC was used by 29% (*n* = 169) of athletes, while 71% were not using HC (Fig. 1).

**Table 3:** Menstrual health characteristics and menstrual dysfunction of non-HC athletes classified into sports disciplines (*n* = 584)

	Endurance	Power	Aesthetic	Weight class	Ball games	Technical	Anti-gravity	*p*-value
Age at menarche, y	13.2 ± 1.5 ^a^(*p* = 0.018)	13.4 ± 1.5	14.3 ± 1.5 ^b^(*p* = 0.018), ^c^(*p* = 0.039), ^d^(*p* = 0.002)	13.8 ± 1.5 ^d^(*p* = 0.025)	13.3 ± 1.6 ^a^(*p* = 0.039)	13.0 ± 1.3 ^a^(*p* = 0.002), ^e^(*p* = 0.025)	13.7 ± 1.5	< 0.001
Gynecological age, y	7.2 ± 4.3 ^e^(*p* = 0.012)	6.9 ± 4.4 ^e^(*p* = 0.004)	5.9 ± 5.1 ^e^(*p* = 0.001)	9.3 ± 3.5 ^a^(*p* = 0.001), ^b^(*p* = 0.012), ^c^(*p* = 0.044), ^f^(*p* = 0.004)	7.8 ± 5.3 ^e^(*p* = 0.044)	8.2 ± 5.7	7.3 ± 3.9	0.032
Stress fractures, *n* (%)	19 (12%)	6 (7%)	4 (9%)	5 (10%)	7 (5%)	3 (3%)	0 (0%)	0.076
Diagnosed eating disorder, *n* (%)	7 (5%)	2 (2%)	0 (0%)	1 (2%)	1 (1%)	0 (0%)	0 (0%)	0.136
Stress urinary incontinence, *n* (%)	25 (16%)	17 (20%)	9 (21%)	14 (29%)	31 (23%)	13 (12%)	2 (33%)	0.089
HC, *n* (%)	44 (28%)	29 (33%)	5 (12%)	15 (35%)	44 (33%)	30 (27%)	0 (0%)	0.059
Current RMC^†^, *n* (%)	70 (64%)	45 (78%) ^a^(*p* = 0.003)	12 (43%) ^d^(*p* = 0.002), ^f^(*p* = 0.003)	20 (65%)	66 (73%)	61 (76%) ^a^(*p* = 0.002)	4 (67%)	0.020
Current PM^†^, *n* (%)	12 (11%)	2 (3%)	4 (14%)	3 (10%)	5 (5%)	6 (8%)	1 (17%)	0.318
*Current MD*^*†*^*, n (%)*								
Primary amenorrhea^†^, *n* (%)	1 (1%)^a*^	1 (2%)	5 (18%)^b*, d*^	0 (0%)	3 (3%)	0 (0%)^a*^	0 (0%)	0.002
Oligomenorrhea^†^, *n* (%)	16 (15%)	5 (9%)	4 (14%)	5 (16%)	12 (13%)	8 (10%)	1 (17%)	0.828
Secondary amenorrhea^†^, *n* (%)	11 (10%)	5 (9%)	3 (11%)	3 (10%)	5 (5%)	5 (6%)	0 (0%)	0.848
*Lifetime MD, n (%)*								
Primary amenorrhea, *n* (%)	10 (7%)^a*^	8 (9%) ^a^(*p* = 0.001), ^d^(*p* = 0.020)	12 (35%) ^b*, c^(*p* = 0.004), ^d*, f^(*p* = 0.001)	6 (13%)	17 (13%) ^a^(*p* = 0.004), ^d^(*p* = 0.001)	2 (2%) ^a*, c^(p = 0.001), ^f^(*p* = 0.020)	1 (17%)	< 0.001
Oligomenorrhea^‡^, *n* (%)	77 (86%)^d*^	34 (74%)	13 (68%)	19 (95%)	44 (68%)	35 (58%)^b*^	3 (75%)	0.001
Secondary amenorrhea^‡^, *n* (%)	45 (50%)	15 (33%)	8 (42%)	9 (45%)	21 (32%)	22 (37%)	2 (50%)	0.298

HC, hormonal contraceptive; MC, menstrual cycle; RMC, regular menstrual cycle; PM, polymenorrhea; MD, menstrual dysfunction; *n* (%), sample size (percentage of). Data are presented in the form: mean ± SD or *n* (%)

^†^Participants who are currently non-HC users, ^‡^Participants who had previously used HC were excluded from the analysis. ^a^different from aesthetics, ^b^different from endurance, ^c^different from ball games, ^d^different from technical, ^e^different from weight class, ^f^different from power. *indicates significant difference with *p* < 0.001

**Fig. 2:**
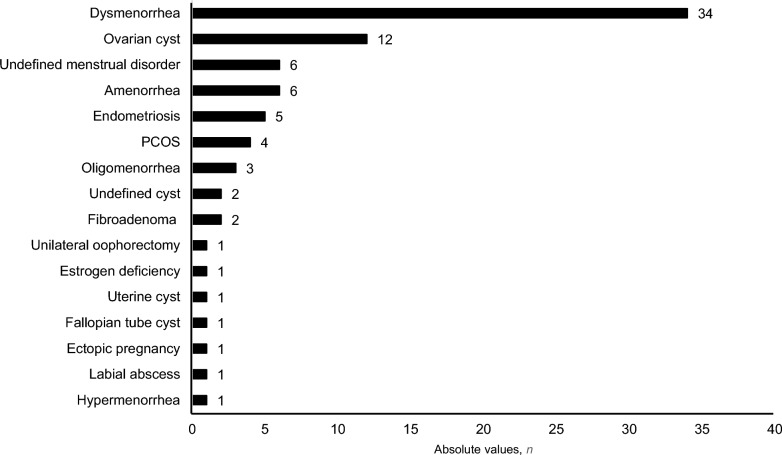
Self-reported currently existing gynecological pre-conditions. PCOS, polycystic ovary syndrome

Overall, 14% of the athletes (*n* = 82) reported existing gynecological pre-conditions (Fig. 2). Furthermore, seven athletes reported pregnancies in the past, while six reported giving birth at least once, and two reported an abortion or miscarriage.

### Menstrual Dysfunction

Nineteen athletes reported not having had their menarche yet, of whom 10 were ≥ 16 y and therefore diagnosed with primary amenorrhea (2%). Two athletes did not report their MC length. In total, 278 of 584 (69%) athletes reported average MC lengths between 25 and 35 days, while 33 (8%) athletes reported polymenorrhea, 51 (13%) oligomenorrhea and 32 (8%) secondary amenorrhea of whom one was pregnant and one was breastfeeding. One athlete explained her current amenorrhea with recently stopping to take the birth control pill, and four athletes offered explanations for current oligomenorrhea or secondary amenorrhea such as recent onset of menarche (*n* = 2), recently stopping to take the birth control pill (*n* = 1), and recent use of the morning after pill (*n* = 1).

The overall lifetime prevalence of primary amenorrhea was 10%. Significant differences between sport disciplines were observed, with a higher lifetime prevalence of primary amenorrhea in athletes competing in aesthetic sports compared to endurance (35% vs. 7%, *p* < 0.001), technical (35% vs. 2%, *p* < 0.001), power (35% vs. 9%, *p* < 0.001), ball games (35% vs. 13%, *p* = 0.001) and weight class (35% vs. 13%, *p* = 0.013) sports. The overall lifetime prevalence of oligomenorrhea was 74%, and for secondary amenorrhea, it was 40% (Table 3). Athletes from technical disciplines had a significantly lower lifetime prevalence of oligomenorrhea than endurance (58% vs. 86%; *p* = 0.004) and weight class (58% vs. 95%; *p* = 0.026) athletes.

There was no significant difference in the lifetime prevalence of secondary amenorrhea between sports disciplines.

**Fig. 3:**
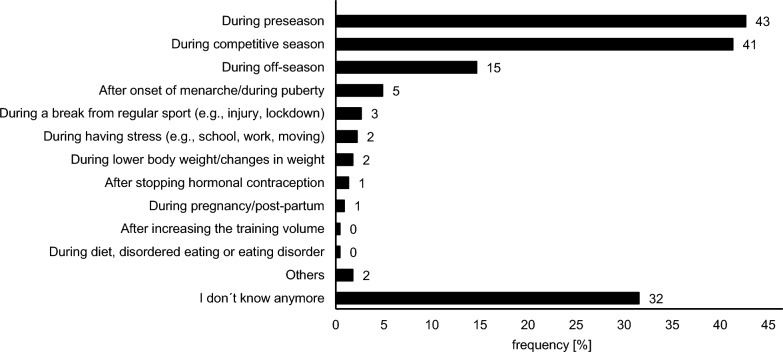
Percentage frequency of the time of menstrual dysfunction, both currently and previously experienced

Regarding the time point when athletes had MD, 43% experienced MD during the preseason, 41% during competitive season, 15% during offseason, 5% after the onset of menarche or during puberty, while 12% reported “other times” and 32% could not remember when MD occurred (Fig. 3). Additional problems that emerged during the time period the athletes suffered from MD were reported by 10% (*n* = 23) and included decreased athlete availability (*n* = 11), tiredness and fatigue (*n* = 4), mental health issues (*n* = 2), mood changes (*n* = 2), impaired gastrointestinal function (*n* = 2), increased states of stress (*n* = 2), decreased cognitive performance (*n* = 1), decreased performance (*n* = 1), decreased training response (*n* = 1), decreased skeletal muscle function (*n* = 1), headaches and migraines (*n* = 1), hyperandrogenemia (*n* = 1), nightly hunger attacks (*n* = 1), and abdominal cramps (*n* = 1).

The binomial logistic regression model revealed a statistically significant relationship between MD and the following variables: gynecological age, diagnosed eating disorder, weekly training volume, BMI, gender distribution of the support team, gender of the main coach, sports discipline, frequency of gynecological health screenings and professional status (χ2 (9) = 75.645, *p* < 0.001). A small amount of variance could be explained by the model, as shown by Nagelkerke’s *R*^*2*^ = 0.260. The overall percentage of accuracy in classification was 80.6%, with a sensitivity of 25.0% and a specificity of 97.1%. Of the nine variables entered into the regression model, two contributed significantly to predicting MD: low gynecological age and no previously diagnosed eating disorder (Table 4).

**Table 4:** Logistic regression analysis of factors associated with menstrual dysfunction in elite female athletes

Variables	Odds ratio	95% confidence interval	*p*-value
Gynecological age (older)	0.817	0.754–0.886	< 0.001
Diagnosed eating disorder (no)	2.681	0.148–0.940	0.037
Weekly training volume (higher)	1.029	0.992–1.068	0.126
BMI (higher)	0.906	0.795–1.032	0.138
Gender distribution of the support team (male dominant)	1.488	0.346–1.305	0.240
Gender of the main coach (female)	1.518	1.690–3.340	0.299
Leanness sports (endurance, aesthetic, weight class, or anti-gravity)	1.252	0.465–1.373	0.417
Frequency of gynecological health screenings (less than once a year)	1.094	0.523–1.596	0.752
Professional status (non-professional)	1.031	0.541–1.964	0.927

BMI, body mass index

The CART model without pruning consisted of up to 15 levels. The model achieved an overall accuracy of 99.0% with a sensitivity of 100% and a specificity of 98.7% based on all previously mentioned variables. The set of variables was incrementally reduced until a lower sensitivity limit of 80 was reached. Generating the tree model only with the three variables gynecological age, BMI, and gender distribution of the support team, it still had a sensitivity of 85.0% and an overall accuracy of 94.5%. Further reducing the set of variables by removing the gender distribution of the support team, the sensitivity reduced to 77.4%.

### Hormonal Contraception

HC users were older than non-HC users (*p* = 0.002), had a higher gynecological age (*p* = 0.010), a higher BMI (*p* = 0.002), were more often in a relationship (*p* < 0.001), had a shorter bleeding duration (*p* < 0.001), perceived less frequent period pain (*p* = 0.045), and took less frequent painkillers during bleeding (*p* = 0.048) (Table 5). Almost three-quarters of the HC users (73%) used combined HC, of which 69% used oral contraceptives (OC) and 6% vaginal rings. Of the progestin-only HCs (27%), 13% used intra-uterine systems, 11% OC, and 2% implants (Fig. 1). Emotional side effects were reported more often to be negative than positive (*p* < 0.001), while practical side effects were reported more often to be positive than negative (*p* < 0.001). The frequency of reporting negative side effects did not significantly differ between progestin-only and combined HC users (28% vs. 18%; *p* = 0.130) (Table 6).

**Table 5:** Participant and gynecological health characteristics divided into HC (*n* = 169) and non-HC (*n* = 415) users

	HC users	Non-HC users	*p*-value
Age, y	21.3 ± 4.2	20.6 ± 5.2	0.002
Gynecological age, y	8.1 ± 4.0	7.6 ± 5.1	0.010
BMI, kg/m^2^	22.5 ± 2.5	21.7 ± 2.3	0.002
Marital status (in a relationship), *n* (%)	118 (70%)	137 (33%)	< 0.001
Weekly training volume, h	17.6 ± 5.9	18.0 ± 7.6	0.791
Bleeding duration of menstruation^†^, d	4.5 ± 1.7	5.1 ± 1.1	< 0.001
Frequency of changing hygiene products, h	4.6 ± 3.1	4.3 ± 3.2	0.081
MC-related symptoms^†^, *n* (%)	33 (19%)	76 (21%)	0.730
Period pain at least during every second menstruation^†^, *n* (%)	94 (55%)	229 (63%)	0.045
Painkiller intake at least during every second menstruation^†^, *n* (%)	32 (19%)	93 (26%)	0.048
Impairment of everyday life due to period pain at least during every second menstruation^†^, *n* (%)	13 (8%)	32 (9%)	0.387
PMS^†^, *n* (%)	22 (13%)	55 (15%)	0.288

BMI, body mass index; PMS, premenstrual syndrome

^†^ Participants who currently do not have any form of amenorrhea

**Table 6:** Frequency and prevalence of self-reported negative and positive side effects among current HC users (*n* = 169)

	Negative side effect	Frequency, *n*	Prevalence, %	Positive side effect	Frequency, *n*	Prevalence, %
**Physical**	Exposure to synthetic hormones	65	39	Reduced dysmenorrhea	48	29
	Weight gain/water retention	15	9	Treatment of menstrual irregularities	25	15
	Irregular/absence of periods	6	4	Reduced bleeding/lighter periods	23	14
	Increased dysmenorrhea	6	4	Treatment of dermatoses (e.g., bad skin)	16	10
	Spotting	5	3	Less frequent/absence of periods	15	9
	Poor skin	4	2	Treatment of (unspecified) MC-related symptoms	8	5
	Headaches/migraines	4	2	No time loss in training	3	2
	Difficulties in muscle gain	3	2	Treatment of endometriosis	2	1
	Pain when inserting	3	2	Stabilization of the hormone balance	2	1
	Gastrointestinal complaints	2	1	No weight changes	2	1
	Longer bleeding period	2	1	No performance changes	2	1
	Cyst formation	2	1	Weight loss	1	1
	Bleeding at unusual times	2	1			
	Weight loss	1	1			
	Increased injury risk	1	1			
	Ectopic pregnancy	1	1			
	Low back pain	1	1			
	Lactose intolerance	1	1			
	Breathlessness/increased HR	1	1			
	Problems with losing weight	1	1			
**Emotional**	Mood changes	31	19	Fewer mood changes	2	1
	Mental health issues (e.g., sadness, depressive symptoms, depression)	8	5	Treatment of PMS	1	1
	Personality changes	2	1			
	Increased states of stress	1	1			
	Tiredness	1	1			
	Less confident with own body	1	1			
**Sexual**	Reduced libido	2	1			
**Practical**	Must be taken regularly/must think of intake	19	11	Contraception	127	77
	Challenges when stopping contraception	2	1	Ability to predict/change menstruation date	73	44
	Concern for long-term health	2	1	Easy to use	29	17
	Lack of information about side effects	1	1	Local effect	5	3
	No 100% contraceptive security	1	1	Long-term option	5	3
	Time elapsed to find an option without side effects	1	1	Free of estrogen	4	2
	Lack of information about alternatives	1	1	Low dose of hormones	3	2
	Permanent foreign body	1	1	Bad reactions to other contraceptive methods	3	2
				Stopping contraception possible at any time	2	1
				No surgical procedure	2	1
				Medical recommendation	2	1
**Other**	No specification	4	2	No specification	4	2
**Non**	No negative side effects reported	35	21			

HC, hormonal contraceptives; HR, heart rate; MC, menstrual cycle; PMS, premenstrual syndrome. Multiple answers were possible

### Gynecological Health Screenings, Menstrual Cycle Tracking and Intentional Weight Changes

Among the elite female athletes ≥ 16 years (*n* = 559), 120 (21%) had never been to a gynecologist. While 64% (*n* = 358) of the athletes had a gynecological screening at least once a year, 14% (*n* = 80) went to the check-up less than once a year. Of the athletes < 16 years (*n* = 25) only one (4%) went to the gynecological check-up less than once a year, while 24 (96%) had never been to a gynecologist. Athletes who went to the gynecological health screening regularly were older (22.2 y vs. 18.5 y; *p* < 0.001), were using HCs (HC 92% vs. non-HC 49%; *p* < 0.001), were in a relationship (relationship 82% vs. single 45%; *p* < 0.001), and in the group of non-HC users were not suffering from MD (no dysfunction 54% vs. dysfunction 39%; *p* = 0.008). Moreover, no differences were observed between athletes with and without MC-related symptoms (64% vs 61%; *p* = 0.324).

Of the 565 athletes who reported their age at menarche, 64% (*n* = 364) tracked their MC. Among non-HC users, athletes who were tracking their MC at the time of the data collection had a significantly lower prevalence of MD (MC tracking 17% vs. no MC tracking 33%; *p* < 0.001). The most common method for tracking was an app (88%), followed by a calendar (7%), smartwatches (3%) and other methods (2%).

**Fig. 4:**
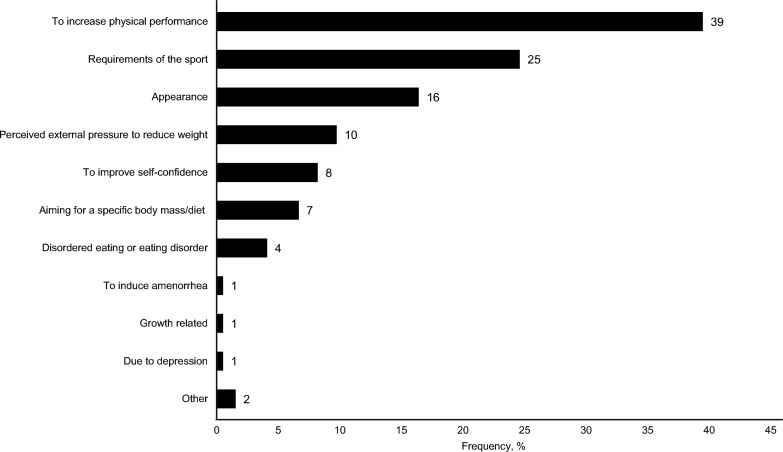
Self-reported reasons for intentional weight changes within the past two years. Weight changes of at least 2 kg weight gain or loss within the past two years

Within the last two years, every third athlete reported to have lost or gained more than 2 kg weight intentionally. The highest proportion was found in athletes from weight class sports (75%), which was significantly higher compared to athletes from endurance (37%; *p* < 0.001), power (29%; *p* < 0.001), aesthetic (33%; *p* < 0.001), ball games (16%; *p* < 0.001) and technical (34%, *p* < 0.001) sports. Athletes from ball games reported significantly fewer intentional weight changes than athletes from endurance (*p* < 0.001), technical (*p* < 0.001), and anti-gravity (67%; *p* = 0.009) disciplines. Athletes who reported intentional weight changes were older (21.3 y vs. 20.5 y; *p* < 0.001), had a higher BMI (22.3 kg/m2 vs. 21.8 kg/m2; *p* = 0.032), were more often professional athletes (38% vs. 28%; *p* = 0.014), were trained more often by a male coach (90% vs. 82%; *p* = 0.010) but did not differ in weekly training volume (18.1 h vs. 17.8 h; *p* = 0.184), competition level (36% international vs. 27% national; *p* = 0.056), training experience in the main sport (11.3 y vs. 11.1 y; *p* = 0.619), and menstrual function (40% MD vs. 33% eumenorrheic; *p* = 0.213). The most common reason for intentional weight changes was “to increase physical performance” (39%), followed by “requirement of the sport” (25%) and “appearance” (16%) (Fig. 4).

## Discussion

The main findings were (1) a higher current and lifetime prevalence of primary amenorrhea in aesthetic sport disciplines, while the lifetime prevalence of oligomenorrhea was higher in endurance disciplines, (2) no significant differences between sport disciplines in the prevalence of current oligomenorrhea and secondary amenorrhea, or the lifetime prevalence of secondary amenorrhea, (3) regular gynecological health screenings and MC tracking were associated with a lower occurrence of MD among elite athletes, as were a higher gynecological age, and surprisingly, a diagnosed eating disorder, whereas intentional weight changes were not, and (4) 29% of elite athletes in Germany used HC, of which 15% used HC as a treatment for MD.

### Menstrual Dysfunction in Different Sports

In all sport disciplines female athletes can be affected by MD. For endurance, aesthetic, and weight class sports, the high prevalence of MD has been documented for many years [2–4]. However, it needs to be highlighted, that athletes in other disciplines, such as ball games, are similarly affected. Although the lowest prevalence of overall MD was observed among athletes in technical disciplines (16%), this is nevertheless two to three times higher than in the general German population [29]. Considering the prevalence irrespective of the type of MD, including primary amenorrhea, secondary amenorrhea, and oligomenorrhea, athletes from aesthetic sports show the highest prevalence (43%), followed by endurance and weight class (each 26%) and, interestingly, ball games (21%). There is a similar tendency for the lifetime prevalence of primary amenorrhea with athletes from aesthetic sports reporting the highest prevalence (35%), followed by anti-gravity (17%), weight class, and ball games (each 13%). For team sports, similar MD prevalences of primary amenorrhea (9%), secondary amenorrhea (8%), and oligomenorrhea (9%) have been reported previously [1]. A possible reason for the high prevalence of MD, especially primary amenorrhea, among athletes from ball games could be premature professionalization. In ball games such as field hockey, the best 14- to 16-year-old players are known to participate in competitions for up to four teams at the same time: the under-16 national team, the state selection team, the club team in their age group and the club team in the next higher age group. As a result, these youth elite players play about 90 competitive matches within a year which equals almost 70 h, excluding hours of training and training matches. In addition to the training and competition load, at this age, requirements at school can further increase the strain [30]. Even with advanced age, the dual career is an additional burden particularly for elite female athletes [31], as professionalization in terms of salary, contracts and facilities, in most female team sports has not progressed to the same level as in male team sports [32]. Meeting the high energy demands resulting from intense exercise activity might be an extra challenge for non-professional elite athletes with a dual career in addition to a full training and competition schedule [33]. As a consequence, REDs may contribute to the observed high prevalences of MD in ball game female athletes, especially at a young age. Therefore, it seems important to recognize that elite female athletes from all sports disciplines, not just from endurance and aesthetic sports, are at risk of developing MD. Additionally, other REDs-related negative health consequences, such as stress urinary incontinence [8], were frequently observed among female athletes. Although no significant differences between sports disciplines were identified, the highest prevalences of stress urinary incontinence were found in anti-gravity sports (33%), weight-class disciplines (29%), and ball games (23%). Previous studies have also reported high prevalences of stress urinary incontinence among female athletes, particularly those with eating disorders[24] or experiencing LEA [34], highlighting that stress urinary incontinence is another important issue that needs to be adequately addressed in female elite sports. Besides athletes and coaches, it seems essential to educate parents about the risk and prevention of MD in the context of elite sports.

### Gynecological Health Awareness

Many elite female athletes neglect their gynecological health, and perceive MD as not being problematic. In Germany, females are advised to visit a gynecologist when they detect an MD, regardless of age [35]. In our study, only 64% of the athletes ≥ 16 years, had regular gynecological screenings, and those who regularly visited the gynecologist showed a significantly reduced prevalence of MD. To date, the authors are not aware of any study that examined the frequency of gynecological screenings among female athletes, but previous research showed that female athletes were unaware of MD and experienced it as a sign of good fitness and as a normal consequence of training rather than a health issue [13, 15]. However, as MD was also normalized in the sports setting by coaches, general practitioners, or other athletes, affected athletes did not see a reason to report MD to gynecologists [15]. Moreover, a recent article revealed, that only 4% of elite athletes in Sweden and Norway, had access to gynecologists within their medical support team [14]. Nevertheless, whenever female athletes consulted gynecologists for medical advice, athletes perceived the recommendations about for example changes in diet or training, often as too general and unspecific for elite sports [13]. Educating gynecologists on the unique physiological requirements of elite sports might help to provide more athlete-oriented gynecological advice. Further, educating female athletes and coaches about the MC, HC, and REDs is important to increase awareness of gynecological health and prevent MD [36]. The athletes’ health may be improved within existing structures if sports medical doctors and team physicians consider gynecological health as an aspect of support in the medical care of female athletes. For this, it is important to educate both sports medical doctors and team physicians regarding gynecological health. Additionally, implementing structures such as obligatory REDs screenings and providing access to elite sports affiliated gynecologists e.g. as part of annual sports medical check-ups may increase the awareness of MD being a first alarm signal for REDs, improve gynecological health, and reduce MD among elite athletes.

Contrary to previous research, the diagnosis of an eating disorder was associated with a lower prevalence of MD. Although eating disorders were previously known as a major cause of LEA and the associated syndrome of REDs [7, 8, 37], it is worth noting that in this study athletes with a diagnosed eating disorder had a lower risk for MD, while athletes without a diagnosed eating disorder had more than 1.5 times higher risk of sustaining an MD. One reason might be that athletes who reported diagnosed eating disorders most likely were in contact with qualified medical staff since eating disorders are usually diagnosed by psychologists or medical doctors [38]. Through the primary treatment of eating disorders, in particular cognitive behavioral therapy [38], specialized professionals may have helped athletes to increase awareness of issues related to eating disorders, including LEA and MD. Access to medical doctors and psychologists seemed to have a positive effect on the occurrence of MD.

In addition, the decision tree approach enabled us to reveal multivariate variables associated with MD. Comparing the logistic regression with the decision tree approach to identify relevant variables associated with MD, it can be stated that using the logistic regression approach is suboptimal because it assumes that a polynomial regression function leads to adequate results, which is not valid in the given case. The regression function mainly adapts to the cases without MD due to skewed distribution in the input data. Furthermore, the statistical indicators (functional weights) for each variable do not take into account the multivariate effects which were detected by using the decision tree as a means of identifying the relevant variables. Due to limited space, the resulting tree cannot be discussed in detail, but at least one interesting rule derived from the tree is presented as an example: the combination of a gynecological age younger than 1.5 years and a support team with equal or female-dominant gender distribution, was always associated with MD. Considering the structures of elite sports, female team physicians or coaches were more likely to work with youth athletes [39]. Additionally, during puberty, MD lasting up to two years is still considered physiological [40]. This is because the hypothalamic-pituitary-ovarian axis is not yet fully developed, leading to common occurrences of anovulatory cycles and variations in MC length ranging from 21 to 45 days [41]. Nevertheless, based on our findings, puberty appears to be essential for preventing MD and negative long-term consequences. Focusing on prevention in young athletes, especially in those who have not yet had their menarche or are in the early stages of puberty, is crucial. Some negative consequences of MD, such as impaired development of peak bone mass, may become irreversible if not addressed early. Therefore, sport federations should create structures, which enable all female athletes, to easily access specialized professionals such as nutritionists, medical doctors or psychologists, which might help to reduce gynecological health risks.

### Hormonal Contraception

In recent years, critical attitudes towards the intake of synthetic hormones have increased [42], leading to a greater skepticism about HC use, yet there is still a lack of specific knowledge on HC in the context of sports. Elite athletes in Germany used HCs less often (29%) compared to the frequency of HC use among elite athletes in Switzerland (45%) [16], in the UK (50%) [22], and in Denmark (57%) [18]. These percentages were similar to that assessed among Australian elite football players (33%) [21]. However, in contrast to the general German population, in which 38% of women between 18 and 49 years use OC [42], OC use is lower amongst elite athletes (18%). From 2018 to 2023, a 19% general decrease of OC use in the German population was observed [42], with non-HC use particularly prevalent among the 16–20 age group [43]. This reduction may be related to increasing skepticism about HC use as almost 60% of the general German population stated that HC had negative consequences for physical and psychological health [42]. Similarly, amongst elite athletes a large number (39%) mentioned “exposure to synthetic hormones” as a negative side-effect. Despite increasing general health awareness and skepticism about synthetic hormones, 14% of the German elite athletes in our study did not know the precise product name of their HC. Similarly 21% of Australian athletes did not know what HC product they were using [21] while revealing poor knowledge about HC function in general [44]. Finally, insufficient knowledge may have resulted in 15% of athletes using HC as a “treatment of menstrual irregularities”. This misbelief has also been detected in athletes from Australia (10%) [21], and the UK (13%) [22]. It could be problematic to use HC as the first and only treatment for MD, as athletes could mistake a withdrawal bleeding for a regular menstrual bleeding, letting them to believe that they have regained a regular MC. To the contrary, without adjusting diet or training volumes, HCs could further mask REDs and the underlying LEA [11, 23]. Therefore, it seems important to educate athletes about the effects of HC in general and its use and associated risks in the context of elite sports.

### Limitations

Since the online questionnaire was distributed through mail and social media it may have been that only interested female athletes with an increased awareness of gynecological health have participated in the survey, which may have introduced bias to the results. As MDs were identified by menstrual cycle length and bleeding pattern, mild forms of MD, such as anovulation, in which an MC is regularly occurring but ovulation is absent, were not detected. In addition, recall bias may have occurred as the current study examined MD with a self-reported online questionnaire that was completed only at a single time. As a result, MD prevalences may likely have been underestimated. Concerning the HC use, we did not ask about the duration of use. As negative side effects are quite common within the first months of use, the duration for which these side effects persist is unclear.. It is therefore possible that the frequency may have been overestimated. A further limitation regarding the assessment of intentional weight changes was, that when we asked about intentional weight changes, we did not distinguish between weight loss and weight gain in the question. In many cases, it was possible to estimate from the context if the athletes had lost or gained weight, as they specified this more precisely in their open-ended answer on reasons for intentional weight gain. If we had been able to differentiate between weight loss and weight gain it may have been that a higher proportion of MD might have been observed in the group of female athletes that lost weight within the previous two years.

## Conclusions

Elite female athletes from all sports disciplines, not just from endurance and aesthetic sports, are at risk of developing MD. In our study, almost 75% of athletes experienced MD in the past and 15% of athletes currently using HC, used HC for “the treatment of menstrual irregularities”. To improve gynecological health, athletes need to recognize MD as a possible symptom of REDs and need to know the effects of HC in general, and therewith associated risks. Thus, educating athletes, coaches, and parents about risk factors and prevention of MD in the context of elite sports seems essential. Further implementing structures such as REDs screenings and providing athletes access to elite sports-affiliated gynecologists, e.g., as part of annual sports medical check-ups, may also improve gynecological health.

## Supplementary Information

Supplementary material 1: Original Questionnaire English Version.

Supplementary material 2: Original Questionnaire German Version.

## Data Availability

The dataset from the current study is available from the corresponding author on reasonable request.

## Abbreviations

MD: Menstrual dysfunction
HC: Hormonal contraceptives
LEA: Low energy availability
REDs: Relative energy deficiency in sport
MC: Menstrual cycle
UK: United Kingdom
GDPR: General data protection regulation
CART: Classification and regression tree
SD: Standard deviation
cm: Centimeter
BMI: Body mass index
y: Years
OC: Oral contraceptives

## Footnotes

1 The manuscripts have been submitted and will be added after publication.
