# Supplementary material for: Prevalence of Menstrual Dysfunction and Hormonal Contraceptive Use Among Elite Female Athletes from Different Sports in Germany
Source: Sports Med Open. 2025 May 7;11:49. doi: 10.1186/s40798-025-00845-6 (PMC12058627; doi:10.1186/s40798-025-00845-6)
Supplement: Supplementary file 1 — Supplementary material 1: Original Questionnaire English Version. [file 40798_2025_845_MOESM1_ESM.pdf]

Supplementary Data Sheet 1: Original Questionnaire English

Personal data

In the following part, we will ask you about general information about yourself, your sport, your level of performance, your training volumes and your sporting environment.
Please try to answer these questions as accurately as possible.
The data will be saved under an anonymous code and it is not possible to draw any conclusions about your person.

First letter of your mother's first name Open-ended
Third letter of your mother's month of birth Open-ended
First letter of your place of birth Open-ended
Last letter of your father's first name Open-ended
First letter of your first name Open-ended
Last letter of your first name Open-ended

Page 1

SAP_1 What is your gender.

- Male
- Female
- Diverse

SAP_2 How old are you?

- Open-ended [Numeric] Years

SAP_3 What is your highest educational level?

- No school-leaving qualification
- Secondary modern school (Hauptschule)
- 10th grade (Realschule)
- High school (Gymnasium)
- Apprenticeship (Ausbildung)
- Polytechnic degree (Fachhochschulabschluss)
- Bachelor, Master, Magister, Diplom (University)
- Doctorate (PhD)
- Others: Open-ended

SAP_4 What is your current marital status?

- Single
- In a relationship, not living together
- In a relationship, living together
- Married
- Divorced
- Widowed
- Other

SAP_5 Which employment relationship best applies to you at this time?

- Sport is my profession.
- I am employed.
- I study.
- I am doing an apprenticeship.
- I go to school.
- Other. Namely: Open-ended

SAP_6 What is your current body height in cm

- Open-ended [Numeric] cm

SAP_7 What is your current body weight in kg?

- Open-ended [Numeric] kg

Page 2

SAP_18 In which federal state do you train?

- Baden-Württemberg
- Bayern
- Berlin
- Brandenburg
- Bremen
- Hamburg
- Hessen
- Mecklenburg-Vorpommern
- Niedersachsen
- Nordrhein-Westfalen
- Rheinland-Pfalz
- Saarland
- Sachsen
- Sachsen-Anhalt
- Schleswig-Holstein
- Thüringen
- I train in another country, namely: Open-ended

SAP_8 In what kind of sport do you participate?

- Badminton
- Track Cycling
- Baseball
- Basketball
- Biathlon
- BMX
- Bobsleigh
- Archery
- Boxing
- Breaking
- Curling
- Ice Hockey
- Figure Skating
- Speed Skating
- Fencing
- Field Hockey
- Football (Soccer)
- Weightlifting
- Golf
- Handball
- Judo
- Canoeing
- Karate
- Track And Field
- Modern Pentathlon
- Mountain Bike
- Nordic Combined

- Para Sport
- Equestrian Sports
- Luge
- Rhythmic Gymnastics
- Wrestling
- Roller Speed Skating
- Rowing
- Rugby
- Shooting
- Swimming
- Sailing
- Short Track
- Skateboard
- Skeleton
- Alpine Skiing
- Ski Cross
- Ski Freestyle
- Cross Country Skiing
- Ski Mountaineering
- Ski Jumping
- Snowboard
- Softball
- Sports Acrobatics
- Sport Climbing
- Road Cycling
- Surfing
- Synchronized Swimming
- Taekwondo
- Tennis
- Table Tennis
- Triathlon
- Gymnastics - Apparatus Gymnastics
- Gymnastics - Trampoline
- Volleyball
- Water Polo
- Water Diving
- Other. Namely: Open-ended

SAP_9 Indicate here the discipline you practice in your sport (if any).

- Open-ended

Page 3

SAP_10 How many years are you participating in your sport?
- Open-ended [numeric] years

SAP_11 What is your current squad status?
- Olympic Squad (OK)
- Perspective Squad (PK)
- Junior Squad 1 (NK 1)
- Junior Squad 2 (NK 2)
- Supplementary Squad (EK)
- National Squad (LK)
- Don't know
- None
- Other. Namely: Open-ended

SAP_12 Please select the highest level that you are currently competing at:
- International (World Cup, European Championships, World Championships,...)
- National (German Championships, National League, Regional League,...)
- State level (e.g. Saxon Championships)
- Other

SAP_13 How many hours of sport do you do on average per week (training and competition)?
  - Open-ended [numeric] hours/ week

SAP_14 How many training sessions do you do on average per week (training and competition)?
  - Open-ended [numeric] sessions/ week

Page 4

SAP_15 What is the gender of your coach with whom you do most of your sessions?

- Male
- Female
- Diverse
- I do not have a coach.

SAP_16 In my support team (coaches, medical staff, physiotherapists) ...

- ... are exclusively women.
- ... are more women than men.
- ... is the ratio quite balanced.
- ... are more men than women.
- ... are exclusively men.

SAP_17 In my training group ...

- ... are exclusively female athletes.
- ... are more female athletes than male athletes.
- ... is the ratio quite balanced.
- ... are more male athletes than female athletes.
- ... are exclusively male athletes.

Gynaecological health

On the following pages we ask you some questions about your menstrual cycle. In addition, we will collect data about your use of hormonal contraceptives. It is important for our study that you answer the upcoming questions as accurately and honestly as possible.
The data will be saved anonymously. It is not possible to draw conclusions about your person.

Page 5

AM_1 How often do you have gynecological appointments (without pregnancy)?
- More than 2x a year
- 2x a year
- 1x a year
- Every 2 years
- Less than every 2 years
- Never

AM_2 Do you have any previous gynecological diseases (ovarian cyst, polycystic ovary syndrome (PCOS), menstrual disorders, severe menstrual pain, ...)?
- Yes
  - If yes, which ones? Open-ended
- No

AM_3 Do you sometimes lose urine unintentionally during sports?
- Yes
- No

AM_4 Have you ever had a stress fracture?
- Yes
  - How many? Open-ended [numeric]
- No

AM_5 Have you ever had a diagnosed eating disorder?
- Yes, currently
- Yes, in the past
- No

AM_6 Have you intentionally lost or gained weight in the last two years?
- Yes
  - Why? Open-ended
- No

AM_7 Have you already had your first period (menarche)?
- Yes
- No

If „yes" at question AM_7 proceed with question AM_8, if „no" finish with this part and proceed with further part

Page 6

AM_8 How old were you when you had your first period (menarche)?

- Open-ended [numeric] years

AM_9 What is the usual amount of days between the first day of period and the first day of your next's period?

- Open-ended [numeric] days
- Currently no applicable, because: Open-ended

AM_10 How many days lasts you period normally?

- Open-ended [numeric] days

AM_11 How often do you change the sanitary products (pad, tampon, menstrual cup, …) during your period?

- Every 5 to 6 hours
- Every 3 to 4 hours
- Every 1 to 2 hours
- Other: Open-ended

AM_12 How often do you have pain or discomfort during your period?

- Every period
- Every second period
- Every third period
- Only in rare exceptional cases
- Never

AM_13 When do you have period pain? When does the pain occur?

- First day only
- 2 days
- Over the entire bleeding period
- Never

AM_14 How often do you use painkillers (e.g. ibuprofen, paracetamol, buscopan or similar) to manage pain during your period?

- Every period
- Every second period
- Every third period
- Only in rare exceptional cases
- Never

AM_15 How often do you miss training or school/study/work because of your period pain?

- Every period
- Every second period
- Every third period
- Only in rare exceptional cases
- Never

AM_16 Do you have pain or discomfort throughout the whole menstrual cycle (i.e. from day 1 of period, until the next period)?

- No
- Yes
    - Which one? Open-ended

If „yes“ at question AM_16 proceed with question AM_17, if „no“ proceed with question AM_18

Page 7

The menstrual cycle can be divided into different phases. To answer the following question, please use the following model as a guide.

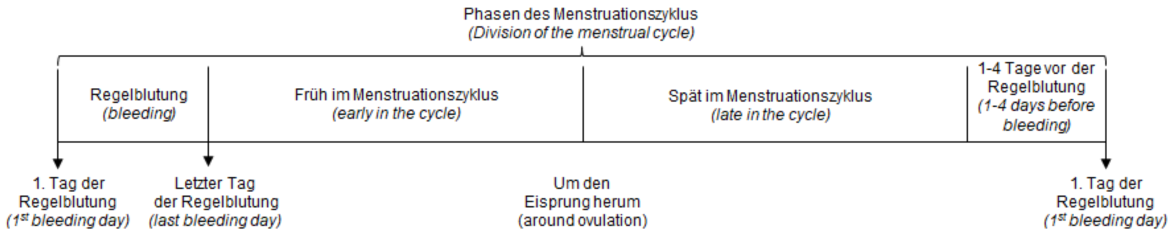

Adapted from Solli et al. 2020

AM_17 When does pain or discomfort occur temporally in the menstrual cycle? (Multiple selections possible)

- Period
- Early in the cycle
- Around ovulation
- Late in the cycle
- 1-4 days before period
- No change
- Do not know

Page 8

AM_18 Have you ever not had a period for more than 5 weeks?

- Yes, currently
- Yes, in the past
- No

If „yes" at question AM_18 proceed with question AM_19, if „no" proceed with question AM_23

<u>Page 9</u>

AM_19 Have you ever not had a period for more than 3 months?
- Yes, currently
- Yes, in the past
- No

AM_20 When was your period irregular (more than 5 weeks apart) or stopped (at least 3 months)? (Multiple selection possible)
- During the competition period
- During training outside the competition period.
- During the non-competitive training period.
- I don't remember in which period.
- Other: Open-ended

AM_21 Did you have additional problems when your periods were irregular or stopped for more than 3 months (more sensitive to injuries, more often ill, ..., ...)?
- Yes
  - Which one? Open-ended
- No

<u>Page 10</u>

AM_23 Do you currently use any contraceptives?
- No
- Yes
  - Which one? Please also specify the name of the preparation.
    - Contraceptive pill: Name/Open-ended
    - Minipill: Name/Open-ended
    - Hormone IUD: Name/ Open-ended
    - Copper IUD: Name/ Open-ended
    - Contraceptive ring: Name/Open-ended
    - Hormone sticks/patches: Name/Open-ended
    - Other, namely: Open-ended

If „yes“ at question AM_23 proceed with question AM_24, if „no“ proceed with question AM_29

Page 11

AM_24 Why do you use hormonal contraceptives (Contraception, better period prediction, treatment of other complaints, ...)

- Open-ended

AM_25 Do you have any symptoms under hormonal contraception?

- Yes
  - Which one? Open-ended
- No

AM_27 What are the advantages of your current contraceptive?

- Open-ended

AM_28 What are the disadvantages of your current contraceptive?

- Open-ended

Page 12

AM_29 Have you used hormonal contraceptives earlier or another hormonal contraceptive in the past?

- No
- Yes
  - Which one? Please also indicate the name of the preparation.
    - Contraceptive pill: Name/Open-ended
    - Minipill: Name/Open-ended
    - Hormone IUD: Name/ Open-ended
    - Copper IUD: Name/ Open-ended
    - Contraceptive ring: Name/Open-ended
    - Hormone sticks/patches: Name/Open-ended
    - Other, namely: Open-ended

If „yes" at question AM_29 proceed with question AM_30, if „no" proceed with question AM_31

Page 13

AM_30 Why did you stopped or changed hormonal contraception?

- Desire to have children
- Intolerance
- weight gain
- Bleeding between periods
- Increased pain
- Mood swings
- Lack of performance progress in sports
- Unsure if it could affect my training adaptations
- Stressful to remember to take
- Other: Open-ended

Page 14

AM_31 Do you currently document your menstrual cycle (e.g. cycle app, cycle diary,...)?

- Yes
    - How? Open-ended
- No

AM_32 Are you currently pregnant/ have you ever been pregnant?

- Yes
    - How many times did you have an abortion? Open-ended [numeric]
    - How many children do you have? Open-ended [numeric]
    - Current
- No
