# Supplementary material for: Prevalence of Menstrual Dysfunction and Hormonal Contraceptive Use Among Elite Female Athletes from Different Sports in Germany
Source: Sports Med Open. 2025 May 7;11:49. doi: 10.1186/s40798-025-00845-6 (PMC12058627; doi:10.1186/s40798-025-00845-6)
Supplement: Supplementary file 2 — Supplementary material 2: Original Questionnaire German Version. [file 40798_2025_845_MOESM2_ESM.pdf]

Supplementary Data Sheet 2: Original Questionnaire German

Angaben zur Person

Im folgenden Teil befragen wir Sie zu allgemeinen Angaben Ihrer Person, Ihrer Sportart, Ihrem aktuellen Leistungsniveau, Ihren Trainingsumfängen und Ihrem sportlichen Umfeld.
Bitte versuchen Sie diese Fragen so genau wie möglich zu beantworten.
Die Daten werden unter einem anonymen Code gespeichert und ein Rückschluss auf Ihre Person ist nicht möglich.

Anfangsbuchstabe des Vornamens Ihrer Mutter Freitext
Dritter Buchstabe des Geburtsmonats Ihrer Mutter Freitext
Anfangsbuchstabe Ihres Geburtsortes Freitext
Letzter Buchstabe des Vornamens Ihres Vaters Freitext
Anfangsbuchstabe Ihres Vornamens Freitext
Letzter Buchstabe Ihres Vornamens Freitext

Seite 1

SAP_1 Bitte geben Sie Ihr Geschlecht an.
- Männlich
- Weiblich
- Divers

SAP_2 Wie alt sind Sie?
    - Freitext/Zahl Jahre

SAP_3 Was ist Ihr höchster Bildungsabschluss?
- Kein Schulabschluss
- Hauptschulabschluss
- Mittlere Reife (Realschule)
- Abitur (Gymnasium)
- Abgeschlossene Ausbildung
- Fachhochschulabschluss
- Bachelor, Master, Magister, Diplom (Hochschule)
- Promotion
- Andere: Freitext

SAP_4 Welchen Familienstand haben Sie aktuell?
- Single
- In einer Beziehung, nicht zusammenlebend
- In einer Beziehung, zusammenlebend
- Verheiratet
- Geschieden
- Verwitwet
- Sonstiges

SAP_5 Welches Beschäftigungsverhältnis trifft derzeit am besten auf Sie zu?
- Der Leistungssport ist mein Beruf.
- Ich bin erwerbstätig.
- Ich studiere.
- Ich mache eine Ausbildung.
- Ich gehe zur Schule.
- Sonstiges. Nämlich: Freitext

SAP_6 Was ist Ihre aktuelle Körpergröße in cm?
- Freitext/Zahl cm

SAP_7 Was ist Ihr aktuelles Körpergewicht in kg?
- Freitext/Zahl kg

<u>Seite 2</u>

SAP_18 In welchem Bundesland trainieren Sie?

- Baden-Württemberg
- Bayern
- Berlin
- Brandenburg
- Bremen
- Hamburg
- Hessen
- Mecklenburg-Vorpommern
- Niedersachsen
- Nordrhein-Westfalen
- Rheinland-Pfalz
- Saarland
- Sachsen
- Sachsen-Anhalt
- Schleswig-Holstein
- Thüringen
- Ich trainiere in einem anderen Land, nämlich: Freitext

SAP_8 Welche Sportart üben Sie aus?

- Badminton
- Bahnradsport
- Baseball
- Basketball
- Biathlon
- BMX
- Bobsport
- Bogenschießen
- Boxen
- Breaking
- Curling
- Eishockey
- Eiskunstlauf
- Eisschnelllauf
- Fechten
- Feldhockey
- Fußball
- Gewichtheben
- Golf
- Handball
- Judo
- Kanusport
- Karate
- Leichtathletik
- Moderner Fünftkampf
- Mountainbike
- Nordische Kombination

- Parasport
- Pferdesport
- Rennrodeln
- Rhythmische Sportgymnastik
- Ringen
- Roller-Speedskating
- Rudern
- Rugby
- Schießen
- Schwimmen
- Segeln
- Shorttrack
- Skateboard
- Skeleton
- Ski Alpin
- Ski Freestyle
- Ski Langlauf
- Ski Mountaineering
- Skispringen
- Snowboard
- Softball
- Sportakrobatik
- Sportklettern
- Straßenradsport
- Surfen
- Synchronschwimmen
- Taekwondo
- Tennis
- Tischtennis
- Triathlon
- Turnsport - Gerätturnen
- Turnsport – Trampolin
- Volleyball
- Wasserball
- Wasserspringen
- Weitere. Nämlich: Freitext

SAP_9 Geben Sie hier die Disziplin an, die Sie in Ihrer Sportart ausüben (falls vorhanden).

- Freitext

Seite 3

SAP_10 Seit wie vielen Jahren üben Sie Ihre Hauptsportart aus?
- Freitext Jahre

SAP_11 Welchen Kaderstatus haben Sie aktuell?
- Olympiakader (OK)
- Perspektivkader (PK)
- Nachwuchskader 1 (NK 1)
- Nachwuchskader 2 (NK 2)
- Ergänzungskader (EK)
- Landeskader (LK)
- Weiß nicht
- Keinen
- Sonstigen. Nämlich: Freitext

SAP_12 Was ist aktuell Ihr höchstes Wettkampfniveau?
- International (Weltcup, Europameisterschaften, Weltmeisterschaften,…)
- National (Deutsche Meisterschaften, Bundesliga, Regionalliga,…)
- Landesebene (z.B. Sächsische Meisterschaften)
- sonstiges

SAP_13 Wie viele Stunden Sport betreiben Sie durchschnittlich pro Woche (Training und Wettkampf)?
- Freitext/Zahl Stunden/ Woche

SAP_14 Wie viele Trainingseinheiten absolvieren Sie durchschnittlich pro Woche (Training und Wettkampf)?
- Freitext/Zahl Einheiten/ Woche

Seite 4

SAP_15 Welches Geschlecht hat Ihr:e Trainer:in, mit dem/der Sie die meisten Einheiten absolvieren?

- Männlich
- Weiblich
- Divers
- Ich habe keine Trainer:in.

SAP_16 In meinem sportlichen Betreuungsteam (Trainer:innen, ärztliches Fachpersonal, Physiotherapeut:innen) …

- … sind ausschließlich Frauen.
- … gibt es deutlich mehr Frauen als Männer.
- … ist das Verhältnis recht ausgeglichen.
- … gibt es deutlich mehr Männer als Frauen.
- … sind ausschließlich Männer.

SAP_17 In meiner Trainingsgruppe …

- … sind ausschließlich Sportlerinnen.
- … gibt es deutlich mehr Sportlerinnen als Sportler.
- … ist das Verhältnis recht ausgeglichen.
- … gibt es deutlich mehr Sportler als Sportlerinnen.
- … sind ausschließlich Sportler.

Angaben zur gynäkologischen Gesundheit

Auf den folgenden Seiten stellen wir Ihnen einige medizinische Fragen zu Ihrem Menstruationszyklus. Darüber hinaus erheben wir Daten über Ihre Nutzung hormoneller Verhütungsmittel. Für unsere Studie ist von entscheidender Bedeutung, dass Sie die kommenden Fragen so genau und ehrlich wie möglich beantworten.
Die Daten werden anonymisiert gespeichert. Ein Rückschluss auf Ihre Person ist nicht möglich.

Seite 5

AM_1 Wie oft werden Sie von einer Frauenärztin/ einem Frauenarzt untersucht (ohne Schwangerschaft)?

- Mehr als 2x im Jahr
- 2x im Jahr
- 1x im Jahr
- Alle 2 Jahre
- Seltener als alle 2 Jahre
- Nie

AM_2 Haben Sie gynäkologische Vorerkrankungen (Eierstockzyste, Polyzystisches Ovar Syndrom (PCOS), Menstruationsstörungen, starke Menstruationsschmerzen, ...)?

- Ja
  - Wenn ja, welche? Freitext
- Nein

AM_3 Verlieren Sie manchmal bei körperlicher Belastung ungewollt Urin?

- Ja
- Nein

AM_4 Hatten Sie jemals einen Ermüdungsbruch (Stressfraktur)?

- Ja
  - Wie viele? Freitext
- Nein

AM_5 Hatten Sie jemals eine diagnostizierte Essstörung?

- Ja, aktuell.
- Ja, in der Vergangenheit.
- Nein

AM_6 Haben Sie in den letzten zwei Jahren absichtlich Gewicht verloren oder zugenommen?

- Ja
  - Warum? Freitext
- Nein

AM_7 Hatten Sie bereits Ihre erste Regelblutung (Menarche)?

- Ja
- Nein

Bei „ja" zu Frage AM_7 weiter bei Frage AM_8, bei „nein" beende diesen Fragebogenpart und weiter mit nächstem Part

Seite 6

AM_8 Wie alt waren Sie, als Sie Ihre Regelblutung (Menarche) zum ersten Mal hatten?
- Freitext/Zahl Jahre

AM_9 Mit welchem Abstand tritt gewöhnlich Ihre Regelblutung auf (jeweils 1. Tag der Blutung)?
- Freitext/Zahl Tage
- Aktuell nicht zutreffend, weil: Freitext

AM_10 Wie viele Tage dauert durchschnittlich Ihre Regelblutung?
- Freitext/Zahl Tage

AM_11 Wie häufig müssen Sie während der Regelblutung Ihre Hygieneartikel (Binde, Tampon, Menstruationstasse, …) wechseln?
- Alle 5 bis 6 Stunden
- Alle 3 bis 4 Stunden
- Alle 1 bis 2 Stunden
- Sonstiges: Freitext

AM_12 Wie oft haben Sie Schmerzen oder Beschwerden während der Regelblutung?
- Bei jeder Regelblutung
- Bei jeder zweiten Regelblutung
- Bei jeder dritten Regelblutung
- Nur in seltenen Ausnahmefällen
- Nie

AM_13 Wann haben Sie Schmerzen oder Beschwerden während der Regelblutung?
- Nur am ersten Tag
- 2 Tage
- Über die gesamte Blutungsdauer
- Nie

AM_14 Wie oft nehmen Sie während Ihrer Regelblutung Schmerzmittel (z.B. Ibuprofen, Paracetamol, Buscopan oder ähnliches) ein?
- Bei jeder Regelblutung
- Bei jeder zweiten Regelblutung
- Bei jeder dritten Regelblutung
- Nur in seltenen Ausnahmefällen
- Nie

AM_15 Sind Ihre Schmerzen so schlimm, dass Sie das Training ausfallen lassen müssen oder nicht zur Schule/ Studium/ Arbeit gehen können?
- Bei jeder Regelblutung
- Bei jeder zweiten Regelblutung
- Bei jeder dritten Regelblutung

- Nur in seltenen Ausnahmefällen
- Nie

AM_16 Haben Sie Schmerzen oder Beschwerden im Verlauf des gesamten Menstruationszyklus (d.h. vom 1. Tag der Blutung, bis zur nächsten Blutung)?

- Nein
- Ja
    - Welche? Freitext

Wenn „ja" zu Frage AM_16 weiter bei Frage AM_17, bei nein bei Frage AM_18

Seite 7

Der Menstruationszyklus kann in unterschiedliche Phasen eingeteilt werden. Für die Beantwortung der folgenden Frage orientieren Sie sich bitte an folgendem Modell.

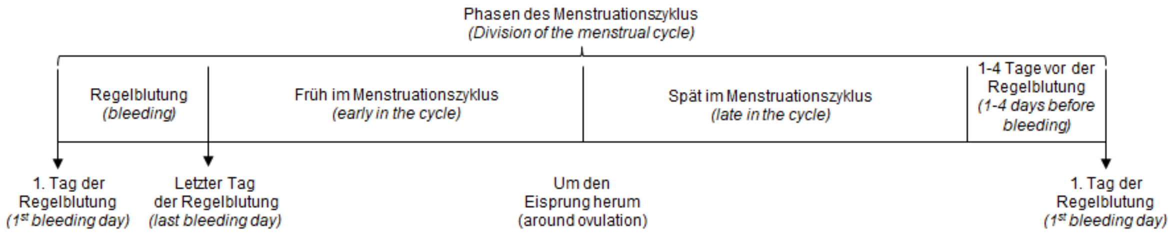

Angepasst nach Solli et al. 2020

AM_17 Wann treten Schmerzen oder Beschwerden zeitlich im Menstruationszyklus auf? (Mehrfachauswahlen möglich)

- Während der Regelblutung
- Früh im Menstruationszyklus
- Um den Eisprung herum
- Spät im Menstruationszyklus
- Ca. 1-4 Tage vor der Regelblutung
- Keine Veränderung
- Weiß nicht

Seite 8

AM_18 Ist es bei Ihnen schon einmal vorgekommen, dass Ihre Regelblutungen einen Abstand von mehr als 5 Wochen hatten?

- Ja, aktuell
- Ja, in der Vergangenheit
- Nein

Bei „ja" zu Frage AM_18 weiter bei Frage AM_19, bei „nein" weiter bei Frage AM_23

Seite 9

AM_19 Ist es bei Ihnen schon einmal vorgekommen, dass Sie mindestens 3 Monate in Folge keine Regelblutung hatten (Schwangerschaft ausgeschlossen)?

- Ja, aktuell
- Ja, in der Vergangenheit
- Nein

AM_20 Wann war Ihre Regelblutung unregelmäßig (Abstand mehr als 5 Wochen) oder hat ausgesetzt (min. 3 Monate)? (Mehrfachauswahl möglich)

- Während der Wettkampfphase.
- Im Trainingsbetrieb außerhalb der Wettkampfphase.
- Während der trainingsfreien Zeit.
- Ich weiß nicht mehr, in welcher Phase.
- Sonstiges: Freitext

AM_21 Hatten Sie zusätzliche Probleme als Ihre Regelblutung unregelmäßig war oder länger als 3 Monate ausgesetzt hat (anfälliger für Verletzungen, häufiger krank, ...)?

- Ja
  - Welche? Freitext
- Nein

Seite 10

AM_23 Nutzen Sie aktuell ein hormonelles Verhütungsmittel?

- Nein
- Ja
  - Welches? Bitte geben Sie auch den Namen des Präparates an.
    - Verhütungspille: Name/Freitext
    - Minipille: Name/Freitext
    - Hormonspirale: Name/Freitext
    - Kupferspirale: Name/Freitext
    - Verhütungsring: Name/Freitext
    - Hormonstäbchen/-pflaster: Name/Freitext
    - Sonstiges: Freitext

Wenn „ja" zu Frage AM_23 weiter bei Frage AM_24, bei nein bei Frage AM_29

Seite 11

AM_24 Aus welchen Gründen nutzen Sie hormonelle Verhütungsmittel (Verhütung, bessere Planbarkeit der Regelblutung, Behandlung von anderen Beschwerden, …)?

- Freitext

AM_25 Haben Sie Beschwerden unter der hormonellen Verhütung?

- Ja
  - Welche? Freitext
- Nein

AM_27 Wo sehen Sie Vorteile Ihres aktuellen Verhütungsmittels?

- Freitext

AM_28 Wo sehen Sie Nachteile Ihres aktuellen Verhütungsmittels?

- Freitext

Seite 12

AM_29 Haben Sie in der Vergangenheit hormonelle Verhütungsmittel oder ein weiteres hormonelles Verhütungsmittel genutzt?

- Nein
- Ja
  - Welches? Bitte geben Sie auch den Namen des Präparates an.
    - Verhütungspille: Name/Freitext
    - Minipille: Name/Freitext
    - Hormonspirale: Name/Freitext
    - Kupferspirale: Name/Freitext
    - Verhütungsring: Name/Freitext
    - Hormonstäbchen/-pflaster: Name/Freitext
    - Sonstiges, nämlich: Freitext

Wenn „ja" zu Frage AM_29 weiter bei Frage AM_30, bei nein weiter bei AM_31

Seite 13

AM_30 Was war der Grund für das Absetzen oder Umstellen der hormonellen Verhütung?

- Kinderwunsch
- Unverträglichkeit
- Gewichtszunahme
- Zwischenblutungen
- Erhöhte Schmerzen
- Stimmungsschwankungen
- Mangelnder Leistungsfortschritt im Sport
- Unsicher, ob es meine Trainingsanpassungen beeinträchtigen könnte
- Stressig, an die Einnahme zu denken
- Sonstiges: Freitext

Seite 14

AM_31 Dokumentieren Sie Ihren Menstruationszyklus (z.B. Zyklus-App, Zyklustagebuch,...)?

- Ja
    - Wie? Freitext
- Nein

AM_32 Waren oder sind Sie schwanger?

- Ja
    - Wie oft hatten Sie einen Schwangerschaftsabbruch?
        - Freitext/Zahl Anzahl
    - Wie viele Kinder haben Sie?
        - Freitext/Zahl Anzahl
    - Aktuell
- Nein
